# Direct Impairment of Vascular Function by Diesel Exhaust Particulate through Reduced Bioavailability of Endothelium-Derived Nitric Oxide Induced by Superoxide Free Radicals

**DOI:** 10.1289/ehp.0800235

**Published:** 2008-12-17

**Authors:** Mark R. Miller, Stephen J. Borthwick, Catherine A. Shaw, Steven G. McLean, Daniel McClure, Nicholas L. Mills, Rodger Duffin, Ken Donaldson, Ian L. Megson, Patrick W.F. Hadoke, David E. Newby

**Affiliations:** 1 Centre for Cardiovascular Science and; 2 Centre for Inflammation Research, University of Edinburgh, Edinburgh, United Kingdom;; 3 Free Radical Research Facility, University of Highlands and Islands, UHI Millenium Institute, Inverness, United Kingdom

**Keywords:** blood vessel, diesel, nitric oxide, particulate, pollution, superoxide

## Abstract

**Background:**

Diesel exhaust particulate (DEP) is a key arbiter of the adverse cardiovascular effects of air pollution.

**Objectives:**

We assessed the *in vitro* effects of DEP on vascular function, nitric oxide (NO) availability, and the generation of oxygen-centered free radicals.

**Methods:**

We assessed the direct vascular effects of DEP (10–100 μg/mL) in isolated rat aortic rings using myography. We investigated NO scavenging and oxygen-centered free radical generation using an NO electrode and electron paramagnetic resonance (EPR) with the Tempone-H (1-hydroxyl-2,2,6,6-tetramethyl-4-oxo-piperidine) spin trap, respectively.

**Results:**

Acetylcholine-induced relaxation was attenuated by DEP (maximum relaxation reduced from 91 ± 4% to 49 ± 6% with 100 μg/mL DEP; *p* < 0.001) but was restored by superoxide dismutase (SOD; maximum relaxation, 73 ± 6%; *p* < 0.001). DEP caused a modest inhibition of relaxation to NO donor drugs, an effect that could be reversed by SOD (*p* < 0.01). At 10 μg/mL, DEP did not affect verapamil-induced relaxation (*p* = 0.73), but at 100 μg/mL DEP inhibited relaxation (*p* < 0.001) by a mechanism independent of SOD. NO concentrations generated by 2-(*N*,*N*-diethylamino)-diazenolate-2-oxide (DEA/NO; 10 μM) were reduced by DEP (100 μg/mL; from 5.2 ± 0.4 to 3.3 ± 0.4 μM; *p* = 0.002). Free radical generation was increased by DEP (10 μg/mL; 9-fold increase in EPR spectra; *p* = 0.004) in a manner that could be attenuated by SOD (*p* = 0.015).

**Conclusions:**

DEP caused oxidative stress through the generation of oxygen-centered free radicals that reduced the bioavailability of endothelium-derived NO without prior interaction with the lung or vascular tissue. These findings provide a mechanism for the adverse cardiovascular effects of particulate air pollution.

Particulate matter (PM) from air pollution is associated with cardiovascular morbidity and mortality (Bhatnagar 2006; Brook et al. 2003; Dockery et al. 1993). These associations are strongest for fine PM [aerodynamic diameter ≤ 2.5 μm (PM_2.5_)] (Miller et al. 2007; Samet et al. 2000), probably due to their large reactive surface area and their potential to penetrate deep into the lung (MacNee and Donaldson 2000). Diesel exhaust emissions are the major source of PM_2.5_ in urban environments, and although they contribute little to the mass of ambient PM, they produce a huge burden in terms of particle numbers (Laden et al. 2000; Peters et al. 1997).

Despite the strength of these epidemiologic associations, the mechanism by which inhaled diesel exhaust particulate (DEP) exerts cardiovascular effects remains to be fully elucidated. *In vivo* and *ex vivo* exposures of animals to a range of environmental and model particles impair vasodilator function (Bagate et al. 2004; Batalha et al. 2002; Folkmann et al. 2007; Hansen et al. 2007; Nurkiewicz et al. 2006, 2008) and encourage the progression of cardiovascular disease (Araujo et al. 2008; Sun et al. 2005, 2008; Suwa et al. 2002). However, the findings from *in vitro* application of PM to isolated vascular tissue have been conflicting, causing both relaxation (Cheng and Kang 1999; Ikeda et al. 1995; Knaapen et al. 2001) and contraction (Li et al. 2005; Tzeng et al. 2003). Furthermore, the *in vitro* effects of PM on endothelium-dependent and -independent vasodilators [e.g., acetylcholine (ACh) and sodium nitroprusside (SNP), respectively] have received surprisingly little attention, and conclusions are difficult to draw because of the diverse range of PM types investigated, different tissues employed, and a lack of appropriate controls.

We have previously shown that inhalation of environmentally relevant concentrations of DEP impairs endothelial and fibrinolytic function in healthy volunteers (Mills et al. 2005; Tornqvist et al. 2007) and in patients with stable coronary heart disease (Mills et al. 2007). In healthy volunteers, forearm relaxation to both ACh and SNP, but not verapamil, was impaired, suggesting that diesel exposure was acting through inhibition of the nitric oxide (NO) pathway. In blood vessels, particularly conduit arteries, NO is a key dilator molecule and of considerable importance both in cardiovascular physiology and in pathophysiology. The selective inhibition of NO-mediated relaxation supports proposals that the vascular effects of DEP are caused by increased generation of oxygen-centered free radicals (Donaldson et al. 2003, 2005; Nel et al. 2001) that can scavenge endogenous NO.

In the present study, we aimed to determine whether DEP directly inhibits endothelium-dependent NO-mediated relaxation, reduces NO bioavailability, and generates oxygen-centered free radicals.

## Materials and Methods

### Preparation of DEP

We prepared fresh DEP suspensions each day (2 mg/mL stock solution) in Krebs buffer (composition in mM: 118.4 NaCl, 25 NaHCO_3_, 11 glucose, 4.7 KCl, 1.2 MgSO_4_, 1.2 KH_2_PO_4_, 2.5 CaCl_2_); the PM was suspended by vortexing for 1 min followed by probe sonication (US70; Philip Harris Scientific, Lichfield, UK) twice for 1 min at 60% power (five cycles), and resonicated (U50; Ultrawave, Cardiff, UK) for 15 min before use.

We measured mean particle diameter by photon correlation spectroscopy using a PS90 particle size analyzer (Brookhaven Instruments Corp., Holtsville, NY, USA) set to the following parameters: 37°C, 90° angle; 1 min run duration; refractive index of PM: 1.5, “real,” and 0, “imaginary”; dust cutoff, 30. We diluted samples 50-fold in Krebs buffer to allow sufficient penetration of laser light through the sample.

### Vascular tissue preparation and protocol

We performed all our experiments according to the Animals (Scientific Procedures) Act 1986 (U.K. Home Office) to ensure that we treated all animals humanely and with regard to the alleviation of suffering. Adult male Wistar rats (250–400 g) were killed by cervical dislocation and the thoracic aorta was dissected free. We cleaned segments (~5 mm length) of connective tissue and mounted them in a multimyograph system (610M; Danish Myo Technology, Aarhus, Denmark) in Krebs buffer bubbled with 5% CO_2_/95% O_2_ at 37°C. A baseline tension of 14.7 mN was gradually applied over 10 min and vessels were allowed to equilibrate for a further 30 min. Preliminary experiments showed that this tension produced optimal contraction and relaxation responses. The data from force transducers were processed by a MacLab/4e analogue-digital converter displayed through Chart software, version 3.4.3 (AD Instruments, Sussex, UK).

We confirmed vessel viability by a contractile response on addition of 80 mM KCl, repeated three times, and then obtained concentration–response curves to phenylephrine (PE; 1 nM to 10 μM) and selected a concentration that produced 80% maximum contraction (EC_80_; 0.1–1 μM) for each individual rat aortic ring. After contraction, we obtained cumulative concentration–response curves for ACh (endothelium-dependent vasodilator; 1 nM to 10 μM), SNP (endothelium-independent NO donor; 0.1 nM to 1 μM), and the calcium channel antagonist verapamil (endothelium- and NO-independent vasodilator; 1 nM to 100 μM). We pretreated all vessels with DEP (100 μg/mL) and/or superoxide dismutase (SOD; 100 U/mL) 20 min before the start of, and throughout, generation of concentration–response curves. We analyzed these four treatment groups in parallel with aortic rings from the same animal. We allowed at least 30 min washout before application of subsequent drugs. Addition of DEP did not affect the pH or osmolarity of the Krebs buffer.

### NO measurement

We measured NO electrochemically with an NO electrode (ISO-NOP; World Precision Instruments, Stevenage, UK) attached to an amplifier (NO meter; ISO-NO Mark II; World Precision Instruments); the electrode was introduced to the cuvette containing 2 mL Krebs buffer with or without DEP (100 μg/mL), pre-warmed to 37°C and stirred continuously at 1,000 rpm. Once we obtained a stable baseline (10–30 min), we calibrated the electrode using 2-(*N*,*N*-diethylamino)-diazenolate-2-oxide (DEA/NO; 100–800 nM) in phosphate buffer at pH 4; DEA/NO undergoes rapid and complete spontaneous decomposition at this pH (Davies et al. 2001). We used Krebs buffer (pH 7.4) for subsequent protocols. We added DEP (100 μg/mL) 2 min before addition of DEA/NO (10 μM) and measured changes in signal for 15 min. Recalibration at the end of the experiment revealed no change in sensitivity. Neither the baseline signal from the microsensor nor the posttreatment calibration to DEA/NO afterward was affected by DEP.

### Electron paramagnetic resonance

We used electron paramagnetic resonance (EPR) to establish oxygen-centered free radical generation from DEP in the absence of tissue. We prepared and incubated solutions with the spin trap, 1-hydroxyl-2,2,6,6-tetramethyl-4-oxo-piperidine (Tempone-H; 1 mM), immediately before the initial measurement. Tempone-H is a highly sensitive spin trap that shows selectivity for superoxide, as well as peroxyl radicals and peroxynitrite, forming a stable product that can be measured by EPR (Dikalov et al. 1997). We used pyrogallol (100 μM) as a positive control to generate superoxide radicals (Taylor et al. 2004). In selected samples, we added SOD (100 U/mL) as a specific scavenger of superoxide anions.

We ensured that samples were kept at 37°C throughout and measurements were taken after 1, 20, 40, and 60 min by drawing 50 μL of sample into a capillary tube (Scientific Laboratory Ltd., Coatbridge, UK) and sealing it with a plug of soft sealant (Cristaseal, VWR International, Lutterworth, UK). We used an X-band EPR spectrometer (Magnettech MS-200, Berlin, Germany) with the following parameters: microwave frequency, 9.3–9.55 Hz; microwave power, 20 mW; modulation frequency, 100 kHz; modulation amplitude, 1,500 mG; center field, 3,365 G; sweep width, 50 G; sweep time, 30 sec; number of passes, 1.

### Drugs and reagents

We obtained Krebs salts from VWR International, and all other drugs from Sigma Ltd. (Poole, UK), with the exception of DEA/NO (Alexis Biochemicals, Nottingham, UK), Tempone-H (Alexis Biochemicals), and DEP [SRM-2975, National Institute of Standards and Technology (NIST), Gaithersburg, MD, USA]. We dissolved and diluted all drugs in Krebs buffer, with the exception of Tempone-H [dissolved in 0.01 M EDTA (final concentration, 0.1 mM) to minimize metal-ion–induced auto-oxidation] and DEA/NO (0.01 M NaOH; final concentration, 0.1 mM). Preliminary experiments confirmed that, in all cases, addition of vehicle alone had no effect on vessel tone or NO electrode measurements.

### Statistical and data analysis

We express all data as mean ± SE, and EPR intensity on an arbitrary scale based on the area under the curve of the first derivative of the first line of the three-line spectrum generated (Taylor et al. 2004) using Miniscope software (version 6.51; Magnettech, Berlin, Germany). We measured maximum responses of the NO microsensor in millivolts and present them as NO concentration (μM) derived from the gradient of the calibration curve. We express vasodilator responses as a percentage of the precontraction to EC_80_ PE, where positive values represent relaxation and 100% relaxation represents a complete abolition of PE-induced tone.

We performed statistical comparisons of concentration–response curves using two-way repeated-measures analysis of variance (ANOVA). Additionally, we calculated maximum vasodilator responses (I_max_) and IC_50_ concentrations (defined as the concentration of vasodilator required to produce 50% relaxation of precontraction tone) following linear regression of individual curves [using GraphPad Prism version 4.0b (Graphpad Software Inc., La Jolla, CA, USA)] and made statistical comparisons using one-way ANOVA, with Bonferroni post hoc tests of selected comparisons where appropriate. We carried out statistical comparisons of NO concentrations using two-way ANOVA and made comparisons of EPR data at 60 min using paired *t*-tests. We considered *p* < 0.05 to be statistically significant.

## Results

### Effect of DEP on vascular function

**Figure 1:**
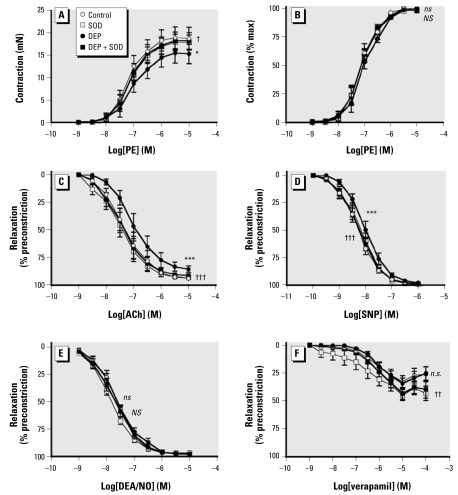
Effect of DEP (10 μg/mL) or vehicle control in the presence (circles) or absence (squares) of SOD (100 U/mL) on contraction and relaxation induced by PE (*A* and *B*), ACh (*C*), SNP (*D*), DEA/NO (*E*), and verapamil (*F*). Vessels were precontracted with EC_80_ PE (0.3–1 μM). Data are mean ± SE (*n* = 7–8 for all). Two-way repeated-measures ANOVA: *p < 0.05, and ***p < 0.001 (*ns*, not significant), control versus DEP; ^†^*p* < 0.05, ^††^*p* < 0.01, and ^†††^*p* < 0.001 (*NS*, not significant), DEP versus DEP + SOD.

**Figure 2:**
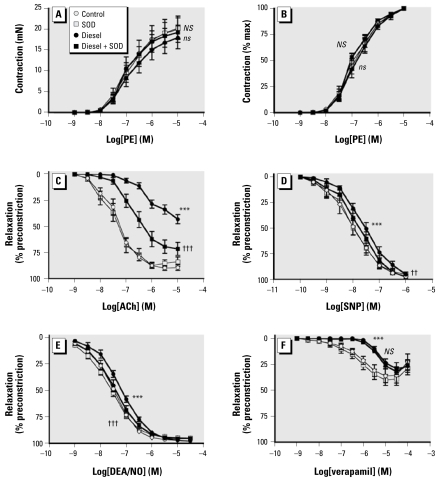
Effect of DEP (100 μg/mL; solid symbols) or vehicle control (open symbols) in the presence (circles) or absence (squares) of SOD (100 U/mL) on contraction and relaxation induced by PE (*A* and *B*), ACh (*C*), SNP (*D*), DEA/NO (*E*), and verapamil (*F*). Vessels were precontracted with EC_80_ PE (0.3–1 μM). Data are mean ± SE (*n* = 6–8 for all). Two-way repeated-measures ANOVA: ***p < 0.001 (*ns*, not significant), control versus DEP; ^††^*p* < 0.01, and ^†††^*p* < 0.001 (*NS*, not significant), DEP versus DEP + SOD.

Photon correlation spectroscopy confirmed that mean diameter of DEP aggregates suspended in Krebs buffer was < 1 μm (0.94 ± 0.01 μm; *n* = 3). Responses to PE were smaller in the presence of 10 μg/mL DEP (*p* = 0.012, two-way ANOVA compared with control; *n* = 7 for all; Figure 1A), although when expressed as a percentage of the maximum contraction, to look at changes in sensitivity to PE, we found no effect from either concentration of DEP (Figures 1B, 2B; Table 1). Consequently, EC_80_ values for PE were similar for DEP- and SOD-treated tissues (*p* = 0.51, one-way ANOVA; *n* = 7 for all).

ACh (1 nM to 10 μM) caused a concentration-dependent relaxation of rat aortic rings. The presence of 10 μg/mL DEP produced a rightward shift in this response (*p* < 0.001, two-way ANOVA; *n* = 7; Figure 1C), lowering log IC_50_ from −7.31 ± 0.22 to −6.99 ± 0.19 in the presence of DEP (*p* = 0.009, one-way ANOVA; *n* = 7; Table 1). Increasing the concentration of DEP to 100 μg/mL markedly inhibited ACh-induced relaxation (Figure 2C), with I_max_ reduced from 91 ± 4% to 49 ± 6% (*n* = 7, *p* < 0.001; Table 1). Coincubation with SOD (100 U/mL) completely reversed the inhibitory effect of 10 μg/mL DEP (*p* < 0.001, two-way ANOVA comparing DEP with DEP + SOD; *n* = 7; Figure 1C) and partially reversed that of 100 μg/mL DEP-induced inhibition of ACh-mediated relaxation (*p* < 0.001; Figure 2C), with I_max_ returning to 73 ± 6% (*p* < 0.001, DEP + SOD vs. DEP alone; *n* = 7; Table 1). SOD (100 U/mL) had no effect on ACh-induced relaxation, or any of the other vasodilators used, in the absence of DEP (*p* > 0.29, two-way ANOVA; *n* = 6–8 for all).

Both concentrations of DEP (10 and 100 μg/mL) caused a modest, but consistent, inhibition of SNP-induced relaxation (*p* < 0.001 for both 10 and 100 μg/mL DEP, two-way ANOVA; *n* = 6–7), without reducing maximum relaxation (*p* = 0.08 and *p* = 0.56, for 10 and 100 μg/mL DEP, respectively, one-way ANOVA; *n* = 6–7; Table 1). The inhibition caused by DEP could be reversed by coincubation with SOD (*p* < 0.001 and *p* = 0.010 for 10 and 100 μg/mL DEP, respectively, two-way ANOVA; Figures 1D, 2D). DEP (100 μg/mL) also produced a similar modest inhibition on relaxation produced by the spontaneous NO donor DEA/NO (*p* < 0.001, two-way ANOVA; *n* = 8) that could be reversed by coincubation with SOD (*p* < 0.001, two-way ANOVA; *n* = 8; Figure 2E, Table 1). DEP had no effect on verapamil-induced relaxation at 10 μg/mL (*p* = 0.73, two-way ANOVA; *n* = 7; Figure 1F) but caused a rightward shift in the concentration–response curve at 100 μg/mL DEP (*p* < 0.001, two-way ANOVAs; *n* = 5) that was unaltered by coincubation with SOD (*p* = 0.20, two-way ANOVA comparing DEP with DEP + SOD; *n* = 5; Figure 2F).

### NO scavenging by DEP

**Figure 3:**
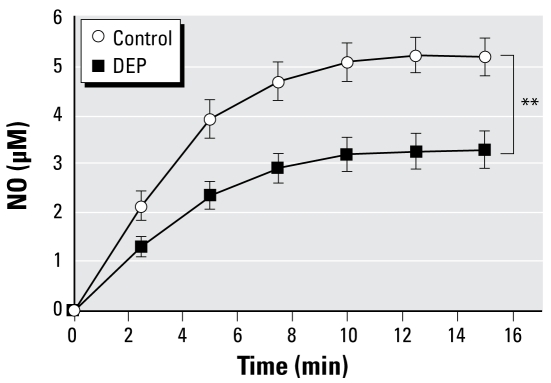
Nitric oxide concentrations generated from the NO donor DEA/NO (10 μM) in the presence or absence of DEP (100 μg/mL). Data are mean ± SE (*n* = 8–10). **p < 0.01, two-way repeated-measures ANOVA.

In Krebs buffer alone, the NO donor DEA/NO (10 μM) caused a time-dependent increase in NO concentration, reaching a plateau of ~5 μM after ~10 min (Figure 3). In the presence of 100 μg/mL DEP, there was a decrease in the NO concentrations from 5.18 ± 0.38 to 3.28 ± 0.38 μM (*p* = 0.002, two-way ANOVA, *n* = 8–10) at 15 min.

### Oxygen-centered free radical generation by DEP

**Figure 4:**
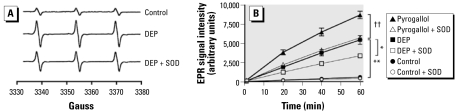
Detection of free radicals from DEP (10 μg/mL) using EPR. (*A*) Sample spectra after 60 min showing the characteristic three-peaked spectrum produced by oxidation of the spin trap Tempone-H (1 mM); DEP (10 μg/mL) caused a large increase in peak amplitude, an effect that was partially reduced by coincubation with SOD (100 U/mL). (*B*) EPR intensity generated by auto-oxidation (control), DEP, or pyrogallol (100 μM) over a 1-hr period, in the presence (open symbols) or absence of SOD (solid symbols). EPR intensity is calculated using the area under the curve of the first derivative of the first line of the three-line spectrum generated. Data are mean ± SE (*n* = 6). Paired *t*-tests: *p < 0.05, DEP versus DEP + SOD; **p < 0.01, control versus DEP; ^††^*p* < 0.01, pyrogallol versus pyrogallol + SOD.

Measurements from control samples (spin trap + drug vehicle) showed a slow time-dependent increase in the characteristic three-line spectrum for a spin adduct with the unpaired electron in the vicinity of a nitrogen atom (i.e., 4-oxo-2,2,6,6-tetra-methylpiperidine-1-oxyl) formed from auto-oxidation of Tempone-H (Figure 4A). The signal increased at a constant rate over the 60-min period (Figure 4B). The EPR signal was enhanced throughout the incubation period in solutions containing 3 μg/mL DEP. EPR intensity was approximately 9-fold higher (*p* = 0.004, paired *t*-test; *n* = 6) in 10 μg/mL DEP suspensions (5,453 ± 577 units) than in controls (624 ± 15 units) after 60 min. SOD (100 U/mL) inhibited the EPR signal from DEP, causing a 39% reduction in signal intensity (3,394 ± 131 units; *p* = 0.015, paired *t*-test; *n* = 6). SOD caused similar effects on signals produced by the superoxide generator pyrogallol (35% reduction; *p* = 0.0015, paired *t*-test; *n* = 6; Figure 4B).

## Discussion

In a comprehensive series of *in vitro* experiments, we have demonstrated that DEP inhibits endothelium-dependent NO-mediated relaxation. This reduced relaxation is consistent with the consumption of NO by DEP-derived oxygen-centered free radicals. These effects were demonstrable in the absence of tissue and without the need for prior interaction with the lung.

### The effect of DEP on vascular function

The mechanism mediating the apparent causal association between air pollution and cardiovascular disease has yet to be fully elucidated. The traditional view is that inhaled PM provoke an inflammatory response in the lung, with subsequent release of prothrombotic and inflammatory cytokines into the circulation (Seaton et al. 1999). Alternatively, the nanometer size of DEP may allow translocation across the respiratory epithelium into the circulation and directly affect the cardiovascular system (reviewed in Oberdorster et al. 2007; Peters et al. 2006). We were therefore interested in the direct effects of DEP on vascular function.

Surprisingly few studies have looked at the effect of PM on vascular tone in isolated blood vessels and have generated contradictory data that, in part, relate to the differences in PM studied. We selected a well-characterized DEP supplied from NIST. Our results showed that the direct addition of DEP to isolated rat aortic rings markedly attenuated endothelium-dependent relaxation induced by ACh. We hypothesized that this inhibitory effect was not confined to the endothelium but would also affect responses to exogenous sources of NO. Consistent with this hypothesis, DEP produced a modest inhibition of vasodilator responses to the endothelium-independent NO generators SNP and DEA/NO. To explore this further, we measured NO release from DEA/NO using a highly selective NO sensor. In the presence of DEP, the levels of NO were markedly decreased, suggesting that DEP scavenges NO and thereby reduces its bioavailability.

Of interest is the greater magnitude of inhibition of relaxation to ACh compared with NO donor drugs. The relatively small inhibition of SNP-induced relaxation is not necessarily surprising considering the intracellular release of NO from this drug (Bates et al. 1991; Kowaluk et al. 1992) that will, to some extent, protect it from extracellular superoxide. However, we observed a similar inhibition of relaxation to DEA/NO even though a substantial proportion of NO release will occur extracellularly in solution (Maragos et al. 1991; Miller et al. 2004). Thus, although scavenging of NO accounts for some of these effects, DEP has additional actions on the vasculature that may include reduced NO generation or perturbation of other endothelial pathways.

The concentrations of DEP used here have few direct cytotoxic effects on endothelial cells (unpublished observations), but they may decrease the release of NO or prostaglandins from the endothelium, and endothelium-dependent hyperpolarizing factors in other blood vessels. The present study uses large-conductance vessels to specifically explore the effect of DEP on the vascular NO pathway. DEP (Hiramatsu et al. 2003; Saito et al. 2002) and urban air PM (Chauhan et al. 2004) decrease the expression of inducible NO synthase, but it is unlikely that changes in protein expression can account for the inhibitory effect of DEP after only 20–30 min of incubation. A more likely hypothesis is that NO synthase activity is altered by DEP, perhaps through altered phosphorylation (Sumanasekera et al. 2007), uncoupling of NO synthase (Knuckles et al. 2008), or up-regulation of the endogenous NO synthase inhibitor asymmetric dimethylarginine (Dvonch et al. 2004). Interestingly, several quinones, which are common components of DEP, decrease the activity of endothelial NO synthase and inhibit the relaxation to ACh, but not SNP (Kumagai et al. 2001; Sun et al. 2006). Work is currently under way to specifically address which components of DEP are responsible for these actions.

Relatively few studies have conducted an in-depth exploration of the mechanism of action of air pollutants on vascular tone through the use of endothelium-dependent and -independent vasodilators (Cheng and Kang 1999; Hansen et al. 2007; Ikeda et al. 1995; Muto et al. 1996; Proctor et al. 2006; Sun et al. 2006). Of these, only three explored the direct effect of DEP on isolated blood vessels, and all have been conducted in rodents. Similar to our own findings, Ikeda et al. (1995) showed that DEP at concentrations > 10 μg/mL inhibited relaxation to ACh and, to a lesser extent, to SNP in rat aortic rings (Ikeda et al. 1995). These findings have been confirmed in rabbit (Muto et al. 1996), but not murine (Hansen et al. 2007), aortic rings. However, none of these studies used an NO-independent vasodilator to ensure that alterations in vascular smooth muscle function did not underlie the reductions in relaxation. Here we show that relaxation induced by the calcium channel antagonist verapamil was unaffected by 10 μg/mL DEP, a concentration that inhibited all other vasodilators tested. However, at the highest concentration of DEP, verapamil-induced relaxation was inhibited by a mechanism that was not reversible by SOD. At such high doses, a more profound effect on the vasculature may occur and result in more widespread tissue effects, including vascular smooth muscle dysfunction.

Our data complement those from previous *in vivo* animal studies showing that inhalation exposure or pulmonary instillation of environmental and model particles is associated with inhibition of endothelium-dependent vasodilators (Bagate et al. 2004; Batalha et al. 2002; Nurkiewicz et al. 2004, 2006, 2008; Sun et al. 2005). Interestingly, vascular responses after controlled exposure of diesel exhaust in humans show a profile of action similar to that seen in our *in vitro* study (Mills et al. 2005). This similarity suggests the possibility of using these combined *in vitro* techniques to screen different PM and manufactured particles.

### Free radical generation by DEP

Free radical generation has been implicated in a wide range of environmental and commercial particles, including asbestos, glass fibers, carbon black, coal dust, quartz, titanium dioxide, and urban PM (Donaldson et al. 2003). DEP has a large reactive surface area and therefore has the potential to generate large amounts of free radicals via the redox-cycling chemicals on its surface. Superoxide is an oxygen-centered free radical that reacts extremely rapidly with NO, lowering its bioavailability and simultaneously producing cytotoxic peroxynitrite. Therefore, loss of endogenous NO due to scavenging by free radicals represents a potential mechanism to link air pollution to the development of cardiovascular diseases (Künzli et al. 2005).

An important finding in our study is that the adverse vascular effects of DEP can be partially prevented by coincubation with SOD. Taken together with the observed reductions in NO concentrations, we suggest the inhibitory effects of DEP are mediated through the generation of superoxide radicals that consume NO. This is a major potential underlying mechanism that links atherosclerosis and cardiovascular disease with air pollution exposure.

PM such as DEP can up-regulate cellular pathways associated with free radical production (Donaldson et al. 2003, 2005), but few studies have looked at the innate ability of DEP to generate free radicals itself. The unstable nature of most free radicals means that they can be quantified only by indirect measures. Compared with other measures of free radicals, EPR is a highly sensitive technique and, by using a spin trap to stabilize the radical of interest, allows measurement of the radical itself, with the characteristics of the free electron providing the signal. Recent improvements in the properties of spin traps have included incorporation of fast reaction rates, stability of radical-trap product, specificity for certain radicals, good signal-to-noise ratio, and a spectrum that allows for characterization and quantification. This has extended the possible applications of this technique and is not affected by the colored solutions such as the intense black of high concentrations of DEP that can restrict some fluorometric or chemiluminescence assays (Aam and Fonnum 2007). Previously, the spin trap 5,5-dimethyl-1-pyrroline-*N*-oxide (DMPO) has been used to demonstrate that DEP gene- rates both superoxide and hydroxyl radicals in the absence of tissue, whereas methanol-washed DEP or pure charcoal produced little in the way of radicals (Sagai et al. 1993). DMPO is a poor spin trap for the detection of superoxide because of its slow reaction rate with this radical and because of the difficulty in separating the peaks in the spectra produced by superoxide from those of hydroxyl radicals.

Here we used the spin trap Tempone-H, which is several orders of magnitude more sensitive for superoxide than for other free radicals (Dikalov et al. 1997). DEP itself generated high concentrations of free radicals in solution. Coincubation with SOD attenuated the amplitude of DEP-induced EPR signals to a similar extent to radical generation by pyrogallol, a compound that generates super-oxide via auto-oxidation in aqueous solution (Marklund and Marklund 1974). The inability of SOD to abolish EPR signals suggests that SOD may be unable to compete with the reaction of superoxide with Tempone-H.

The scavenging of DEP-derived free radicals by SOD complements its ability to reverse the inhibitory effect of DEP on NO-mediated vascular responses. This is the first time that a superoxide-selective spin trap has been used to clarify the free radicals generated by DEP. The results highlight the innate potential of these particles to generate oxygen-centered free radicals. Importantly, these results show that combustion-derived nanoparticles do not necessarily require the recruitment of inflammatory cells from the lung to cause oxidative stress and damage.

### Limitations

We have shown that DEP can directly impair vascular function without prior interaction with lung tissue. PM can cross barriers into the brain or circulation (Berry et al. 1977; Chen et al. 2006; Kreyling et al. 2002; Kwon et al. 2008; Nemmar et al. 2001, 2002; Oberdorster et al. 2002; Takenaka et al. 2001) and may directly interact with vascular tissue to cause effects similar to those described here. However, we acknowledge that we have employed high *in vitro* concentrations to achieve these effects, and it is unclear how such observations relate to the anticipated exposure derived from the inhalation of ambient air pollution. Conversely, *in vitro* effects may underestimate free-radical–initiated actions due to the absence of oxidative stress amplification by inflammatory cells, or the depletion of antioxidant defenses associated with cardiovascular disease. Nevertheless, the similarity of our *in vitro* findings with those of clinical exposure studies (Mills et al. 2005; Tornqvist et al. 2007) suggests that, despite the necessity for high particle concentrations, these techniques are predictive of their cardiovascular toxicology. The use of these *in vitro* techniques could provide an initial step to determine the cardiovascular toxicity and potency of different environmental and engineered PM as well as the effects of fuel modification and particle trap technology.

## Conclusions

DEP directly inhibits vascular relaxation to endothelium-dependent vasodilators through the consumption of NO by oxygen-centered free radicals, such as superoxide. These results demonstrate the innate ability of the PM in air pollution to impair vascular function and highlight the need to identify the toxic components of combustion-derived nanoparticles.

## Figures and Tables

**Table 1:** Effect of DEP and SOD on aortic ring contraction and relaxation (mean ± SE).

	PE (*n* = 7)		ACh (*n* = 7)		SNP (*n* = 6–7)		DEA/NO (*n* = 8)		Verapamil (*n* = 5–7)	
	Log EC_50_	Max. contraction	Log IC_50_	I _max_ (%)	Log IC_50_	I _max_ (%)	Log IC_50_	I _max_ (%)	Log IC_50_	I _max_ (%)
10 μg/mL DEP										
Control	−7.13 ± 0.14	18.75 ± 2.49	−7.31 ± 0.22	95.5 ± 0.9	−8.33 ± 0.12	98.1 ± 0.9	−7.71 ± 0.11	97.9 ± 0.4	−5.92 ± 0.48	38.3 ± 6.9
SOD	−7.14 ± 0.10	17.89 ± 1.66	−7.52 ± 0.24	93.9 ± 2.0	−8.22 ± 0.12	100.8 ± 1.1	−7.85 ± 0.05	96.9 ± 0.9	−6.44 ± 0.48	46.8 ± 5.0
DEP	−7.04 ± 0.13	15.30 ± 2.16	−6.99 ± 0.19*	86.1 ± 2.8**	−8.02 ± 0.12*	97.7 ± 0.3	−7.80 ± 0.16	98.6 ± 0.4	−6.19 ± 0.05	30.0 ± 5.3
DEP + SOD	−7.12 ± 0.10	18.21 ± 1.85	−7.41 ± 0.20 ††	91.8 ± 1.7	−8.30 ± 0.11	99.5 ± 0.9	−7.68 ± 0.11	99.0 ± 0.7	−6.08 ± 0.13	42.7 ± 7.3
100 μg/mL DEP										
Control	−6.88 ± 0.19	19.99 ± 3.18	−7.33 ± 0.08	91.2 ± 3.7	−7.04 ± 0.21	100.0 ± 2.4	−7.48 ± 0.06	97.9 ± 0.3	−6.57 ± 0.25	37.7 ± 5.6
SOD	−6.90 ± 0.12	20.25 ± 2.47	−7.51 ± 0.17	86.2 ± 3.6	−7.02 ± 0.15	97.0 ± 2.5	−7.62 ± 0.08	95.5 ± 0.7	−6.37 ± 0.12	36.8 ± 5.9
DEP	−6.84 ± 0.14	17.97 ± 2.66	−6.04 ± 0.16***	48.5 ± 6.2***	−6.52 ± 0.14	101.0 ± 1.4	−7.19 ± 0.06**	97.9 ± 1.1	−5.37 ± 0.06***	28.8 ± 6.1
DEP + SOD	−6.98 ± 0.07	19.24 ± 1.70	−6.66 ± 0.13††	72.9 ± 6.0†	−7.25 ± 0.60	101.7 ± 2.5	−7.45 ± 0.10†	96.1 ± 1.2	−5.45 ± 0.02	29.6 ± 4.3

* *p* < 0.05’

** *p* < 0.01, and

*** *p* < 0.001, control versus DEP

† *p* < 0.05, and

†† *p* < 0.01, DEP versus DEP + SOD, by Bonferroni post hoc test after one-way ANOVA.
